# Genetic basis of pyrethroid resistance in a population of *Anopheles arabiensis*, the primary malaria vector in Lower Moshi, north-eastern Tanzania

**DOI:** 10.1186/1756-3305-7-274

**Published:** 2014-06-19

**Authors:** Johnson Matowo, Christopher M Jones, Bilali Kabula, Hilary Ranson, Keith Steen, Franklin Mosha, Mark Rowland, David Weetman

**Affiliations:** 1 Kilimanjaro Christian Medical University College (KCMUCo), Moshi, Tanzania; 2 Liverpool School of Tropical Medicine (LSTM), Liverpool, UK; 3 London School of Hygiene and Tropical Medicine (LSHTM), London, UK; 4 Pan-African Malaria Vector Control Consortium (PAMVERC), Moshi, Tanzania; 5 National Institute for Medical Research (NIMR), Tukuyu, Tanzania

**Keywords:** *Anopheles arabiensis*, Genes, Microarrays, Permethrin, Resistance, Transcription

## Abstract

**Background:**

Pyrethroid resistance has been slower to emerge in *Anopheles arabiensis* than in *An. gambiae s.s* and *An. funestus* and, consequently, studies are only just beginning to unravel the genes involved. Permethrin resistance in *An. arabiensis* in Lower Moshi, Tanzania has been linked to elevated levels of both P450 monooxygenases and β-esterases. We have conducted a gene expression study to identify specific genes linked with metabolic resistance in the Lower Moshi *An. arabiensis* population.

**Methods:**

Microarray experiments employing an *An. gambiae* whole genome expression chip were performed on *An. arabiensis*, using interwoven loop designs. Permethrin-exposed survivors were compared to three separate unexposed mosquitoes from the same or a nearby population. A subsection of detoxification genes were chosen for subsequent quantitative real-time PCR (qRT-PCR).

**Results:**

Microarray analysis revealed significant over expression of 87 probes and under expression of 85 probes (in pairwise comparisons between permethrin survivors and unexposed sympatric and allopatric samples from Dar es Salaam (controls). For qRT-PCR we targeted over expressed ABC transporter genes (*ABC* ‘2060’), a glutathione-S-transferase, P450s and esterases. Design of efficient, specific primers was successful for *ABC* ‘2060’and two P450s (*CYP6P3*, *CYP6M2*). For the *CYP4G16* gene, we used the primers that were previously used in a microarray study of *An. arabiensis* from Zanzibar islands. Over expression of *CYP4G16* and *ABC* ‘2060’ was detected though with contrasting patterns in pairwise comparisons between survivors and controls. *CYP4G16* was only up regulated in survivors, whereas *ABC* ‘2060’ was similar in survivors and controls but over expressed in Lower Moshi samples compared to the Dar es Salaam samples. Increased transcription of *CYP4G16* and *ABC* ‘2060’ are linked directly and indirectly respectively, with permethrin resistance in Lower Moshi *An. arabiensis*.

**Conclusions:**

Increased transcription of a P450 (*CYP4G16*) and an ABC transporter (*ABC* 2060) are linked directly and indirectly respectively, with permethrin resistance in Lower Moshi *An. arabiensis*. Our study provides replication of *CYP4G16* as a candidate gene for pyrethroid resistance in *An. arabiensis*, although its role may not be in detoxification, and requires further investigation.

## Background

Vector control programmes employing ITNs and IRS are a mainstay of malaria control [1,2]. Continuous usage of insecticides under such malaria control programmes and/or agricultural application of insecticides has resulted in the development of resistance in major malaria vector species [3-7]. Anopheline mosquito populations in many parts of Africa have developed resistance to pyrethroids [8], which are widely used for indoor residual spraying (IRS) and the only insecticide class available for insecticide treated nets (ITNs). Pyrethroids have been shown to pose very low health risks to humans and other mammals, but are toxic to insects and knock them down (kill them), even at very low doses [9].

Insecticide resistance is considered a major threat to the continued success of IRS and ITNs, with reduction in efficacy of pyrethroids being a particular concern [8,10]. The various mechanisms by which mosquitoes are thought to develop resistance to insecticides include metabolic resistance, target-site resistance, reduced penetration and behavioural avoidance. However, metabolic resistance and target-site resistance are major mechanisms assumed to be responsible for insecticide resistance [11] and are known to contribute to pyrethroid resistance in malaria vectors. While target-site resistance occurs when the site of action of insecticide is altered such that the insecticide no longer binds effectively, metabolic resistance involves over-expression of enzymes capable of detoxifying insecticides or amino acid substitutions within these enzymes, which alter the affinity of the enzyme for the insecticide [12]. The most common target site resistance mechanism constitutes two point mutations at amino acid position 1014 of the voltage-gated sodium channel gene, resulting in either a leucine-phenylalanine (L1014F) [13], or a leucine-serine (L1014S) substitution [14].

Detoxification enzymes include glutathione-S-transferases (GSTs), esterases and P450 monooxygenases. GST-based DDT resistance by breaking down DDT to non-toxic products is common in a number of anopheline species including *An. gambiae*[15-17]. It has been suggested that GSTs may play a role in pyrethroid resistance by detoxifying lipid peroxidation products induced by pyrethroids and/or by protecting from insecticide exposure induced oxidative stress [18]. GSTs might also confer a secondary role in pyrethroid resistance by sequestering the insecticide, hence reducing the total *in vivo* concentration of insecticide [19]. More recently, the study of Riveron *et al.,* 2014 [20] revealed a more direct role played by GSTs in conferring insecticide resistance in malaria vectors.

Elevated levels or activity of esterase enzymes which hydrolyze ester bonds or sequester insecticides is one of the most common metabolic resistance mechanisms in organophosphate (OPs) resistant mosquito species. Malathion (OP) resistance in *Anopheles culicifacies* and *Anopheles stephensi* has been associated with an altered form of esterase that specifically metabolizes the molecule at a much faster rate than that in susceptible strains [21,22].

Cytochrome P450-dependent monooxygenases are an important and diverse family of enzymes involved in the metabolism of numerous endogenous and exogenous compounds. Cytochrome P450 belongs to six families and increased transcription of genes belonging to the CYP4, CYP6, and CYP9 has been observed in various insecticide-resistant species [23]. Microarray-based approaches have identified three candidate P450 genes, CYP6M2, CYP6P3 and CYP6Z2 that were found to be repeatedly over-produced in pyrethroid resistant populations of *An. gambiae *[24-26]. All of these genes encode for enzymes that are able to bind to type I and type II pyrethroids but only CYP6P3 and CYP6M2 were shown to metabolize the insecticides [27,28]. More recently, some studies demonstrated that CYP6M2 is also capable of metabolizing the organochlorine insecticide DDT in *An. gambiae*, hence demonstrating the first evidence for a metabolic cross-resistance in malaria vectors [29].

Both metabolic resistance and target-site resistance have been documented in *An. arabiensis,* one of the dominant vector species of malaria in sub-Saharan Africa [30,31] and in some areas such as the Great Rift Valley in East Africa, *An. arabiensis* is the predominant malaria vector species [32]. To date, the *kdr* mutations have been reported in several countries including Uganda [33], Sudan [34,35], Cameroon [36], and Tanzania [37-39]. In addition to target site resistance, metabolic resistance has also been documented in different countries [40-42].

The study of dynamics of insecticide resistance in Lower Moshi *An. arabiensis* from 2009 to 2013 [39] has shown that the population has developed resistance to all pyrethroids tested (permethrin, deltamethrin and lambdacyhalothrin) with the presence of L1014F mutation at very low frequency. Tanzania has been scaling-up the use of pyrethroid-based LLINs which reached its universal coverage under Universal Coverage Campaign [43]. Therefore, higher pyrethroid resistance is expected following higher selection pressure on this Lower Moshi *An. arabiensis* population and it is likely that pyrethroid resistance will spread to other areas in the country. The previous study by Matowo *et al.,* 2010 [44] had revealed significantly higher levels of oxidase and β-esterase enzymes in wild Lower Moshi *An. arabiensis* than in a laboratory reference strain. However, the genes responsible for elevated levels of these detoxification enzymes remained unknown, although over-expression of a few detoxification genes has been documented in *An. arabiensis* populations in Hai district, adjacent to Lower Moshi. [45]. Therefore, the aim of this study was to explore and identify specific genes involved in metabolic resistance to permethrin in Lower Moshi populations of *An. arabiensis* from Lower Moshi.

## Methods

### Mosquito sample collection and identification

Collections were conducted in the rainy season between May and June 2011 in Mabogini village (37°21′E, 3°24′S), Mabogini is an agricultural area of about 15 km south of Moshi town, in which rice is cultivated in two growing seasons, the main season in which rice is sown is mid-June with irrigation from June to October, and a second season of sporadic cultivation from September to February. The rice paddies thus provide breeding sites for *Anopheles arabiensis,* the predominant malaria vector in the area, throughout much of the year.

The larvae were collected from two specific localities, Mbugani (37°22' E, 3°25'S) and Harusini (37°21'E, 3°23'S) in Mabogini village which are 4 km. apart. Additional larval collections were made from Mbugani in August 2012.

Anopheline larvae were also collected from temporary breeding habitats in Ilala (Gerezani) (39°16' E, 6°49'S) and Kinondoni (Kawe) (39°13' E, 6°43'S) in Dar es salaam about 700 km from Moshi, in August and October 2011.

The field-collected anopheline larvae were transferred into an insectary and reared to adults. Emergent adults were split by sex before mating could occur and fed with 10% sugar solution.

Virgin females were preferred following the previous whole-body microarray experiments [46] that detected large transcriptional changes in mated females where the number of genes differentially expressed increased with time post mating.

At 3 days-old, adult females from Mbugani were exposed to 0.75% permethrin for one hour using a standard WHO tube bioassay procedure [47] with mortality scored 24 hours after the exposure period. Adult mosquitoes from Harusini, Moshi and Dar es Salaam were not exposed to insecticide. At their fourth day post emergence, all unexposed (control) females and the survivors (resistant mosquitoes) were killed in 75% ethanol. A hind leg was removed from each mosquito and stored over silica gel in an individually-labelled tube before placing the remainder of the specimens in RNA later in an Eppendorf tube. DNA was extracted from the legs that were removed from each specimen for species identification.

Species identification focused on the most prevalent local vectors of *An. gambiae sensu lato*; *An. arabiensis* and *An. gambiae s.s*. Mosquitoes were identified to species level using standard PCR method [48]. Only those identified as *An. arabiensis* were further analysed in this study.

## Whole genome microarrays

### Experimental design

The study was designed to comprise of two microarray experiments. The first involved three groups of samples collected in 2011 from Mbugani (insecticide exposed, selecting for the 24% and 54% of the most resistant females [39] and unexposed) and Harusini (unexposed). Although initially suspected that Harusini might display a lower resistance level than Mbugani, its close proximity made predictions ambiguous and only results from hybridizations of Mbugani permethrin-selected against Mbugani unexposed and Mbugani permethrin-selected against Harusini unexposed were used. In the second experiment, to investigate temporal repeatability of results we repeated the experiment from Mbugani (permethrin-selected against. unexposed) using a collection from 2012. In this second experiment a pyrethroid-susceptible sample from Kinondoni and Ilala in Dar-es-Salaam [40] was also included, primarily to permit an allopatric comparison with the population from Mbugani (insecticide exposed that selected for the 54% of the most resistant females [39]. However, we found that comparisons involving the Dar-es-Salaam sample yielded very little additional resolution for the analysis, which coupled with suspected technical errors in some of the hybridizations led to their exclusion.

2011: Mbugani permethrin selected vs Mbugani unexposed

2011: Mbugani permethrin-selected vs. Harusini unexposed

**Figure 1:**
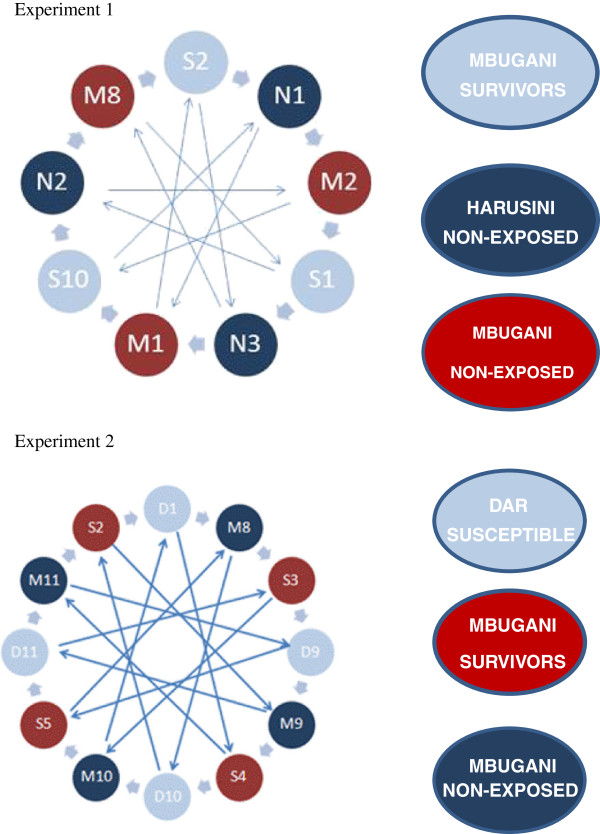
Interwoven microarray experimental loop design for experiment 1 and 2.

2012: Mbugani permethrin-selected vs. Mbugani unexposedComparisons in experiment 1 consisted of three independent biological replicates while those of experiment 2 consisted of four independent biological replicates (Figure 1); and two technical repeats of dye swaps to control for dye bias. Hybridization and analysis followed an interwoven loop design.

To be judged significant a probe was required to (a) exhibit a false discovery rate-corrected probability of q < 0.05 in all three comparisons, and (b) exhibit a consistent directionality in expression, i.e. either Mbugani permethrin-selected groups > unexposed groups or Mbugani permethrin-selected groups < unexposed groups in all three comparisons. Owing to the use of within population (i) selected vs. (ii) unexposed comparisons, the magnitude of differential expression is expected to be relatively low, because (i) is a subset of (ii) [20] and therefore no fold-change threshold was applied as a significance criterion.

### RNA extraction and labelling

Both four day old unfed unmated female mosquitoes that survived one hour exposure to 0.75% permethrin, held for 24 hours post–exposure and unexposed mosquitoes were killed using 70% absolute ethanol and stored in RNAlater® at -20°C. Total RNA was extracted from batches of ten mosquitoes using the RNAqueous^*R*^-4PCR Kit (Ambion) according to manufacturer’s instructions and treated with DNase I (Qiagen). The quality and quantity of the RNA was checked using a using a Nanodrop spectrophotometer (Nanodrop Technologies, Wilmington, DE, USA). and Bioanalyser (Agilent Technologies, Stratagene, USA) respectively. Extracted mRNA was dye-labelled using the Low Input Quick Amp Labelling Kit (Agilent Technologies, Stratagene, USA) according to manufacturer’s instructions. Labelling was repeated for samples that had a low cDNA yield (<15 ng/μl) and poor dye incorporation (<6.0 pmol/μl of each dye).

The labelled samples were hybridized using a Gene Expression Hybridization Kit (Agilent Technologies). Washing, scanning and feature extraction were performed according to the manufacturer’s recommendations.

### Microarray analysis

Analysis of the microarray data was performed on corrected spot intensities using the LIMMA 2.4.1 software package [49] for R 2.3.0. Normalization of signal intensities was followed by ANOVA F-test using the MAANOVA package in R [50]. Microarray data are available in the ArrayExpress database (http://www.ebi.ac.uk/arrayexpress) under accession number E-MTAB-2513 (SURV vs UNEXPOSED loop design) and E-MTAB-2514 (SURV vs DAR loop design).

### Quantitative real time PCR (qPCR)

Technical repeatability of microarray results for a subset of candidate genes was assessed using Quantitative PCR using the cDNA synthesised from the same RNA samples used in the microarray experiments. The samples were quantified using a Nanodrop spectrophotometer (Nanodrop Technologies, Wilmington, DE, USA). cDNA was synthesised from ~0.5-1 μg of RNA using oligo(dT)20 (50 μM) and SuperScript III (200U) (Invitrogen) and purified through a DNA-binding column (Qiagen). The quality and quantity of cDNA was measured using the Nanodrop spectrophotometer (Nanodrop Technologies, Wilmington, DE, USA) and the cDNA samples were stored at -70°C until further use.

Primers for qPCR were designed using NCBI primer BLAST (http://www.ncbi.nlm.nih.gov/tools/primer-blast/) by using Xm codes from Vector Base. Serial dilutions of cDNA were used for standard curve production to determine PCR efficiency and specificity of the primer pairs. The qPCR reactions were performed using the Agilent MXPro Real-Time PCR detection system (Agilent Technologies, Stratagene, USA).

A total volume of 20 μl contained 10 μl Brilliant III SYBR Green, 300 nM of primers, 2.5 μl of cDNA (1:10 dilution) and the total volume made up with sterile-distilled water. The cycling conditions for all primer sets consisted of 95°C for 15 minutes followed by 40 cycles of 60°C for 10s, 95°C for 30s, 95°C for 1 minute, 55°C for 30s and 95°C for 30s. Three biological replicates were run for each sample on a plate. Each gene was measured in at least six real-time PCR runs (replicate reactions/biological repeats). The real-time PCR (qPCR) reactions were run along with two normalising ‘control’ genes, ribosomal S7 and elongation factor EF1. Primer pairs exhibiting a linear relationship between Ct values and template concentration in standard curves, and high PCR amplification efficiency were chosen for further analysis.

### Data analysis of gene expression

Outlier data points were identified and excluded from analysis based on obvious deviations in both the normal shape of amplification curves and the Ct values of other repeated observations (biological triplicates). The mean of the threshold cycles (Ct) for each gene were normalised against the average values for ribosomal S7 and elongation factor EF1. To compare gene expression between treatments, we calculated the ΔΔCt [51]. The fold-change in gene expression for each target gene, normalized to the ribosomal S7 and elongation factor EF1 relative to a susceptible strain of *An. arabiensis* from Dar es Salaam, was calculated according to the 2^-ΔΔCt^ method incorporating PCR efficiency [51]. Basic data analysis (regression and t-tests were performed in Excel. with p < 0.05 used to assess significant difference between treatments for the t-tests.

### Ethical considerations

The study received ethical clearance from the KCMC Research Ethics Committee.

## Results

**Figure 2:**
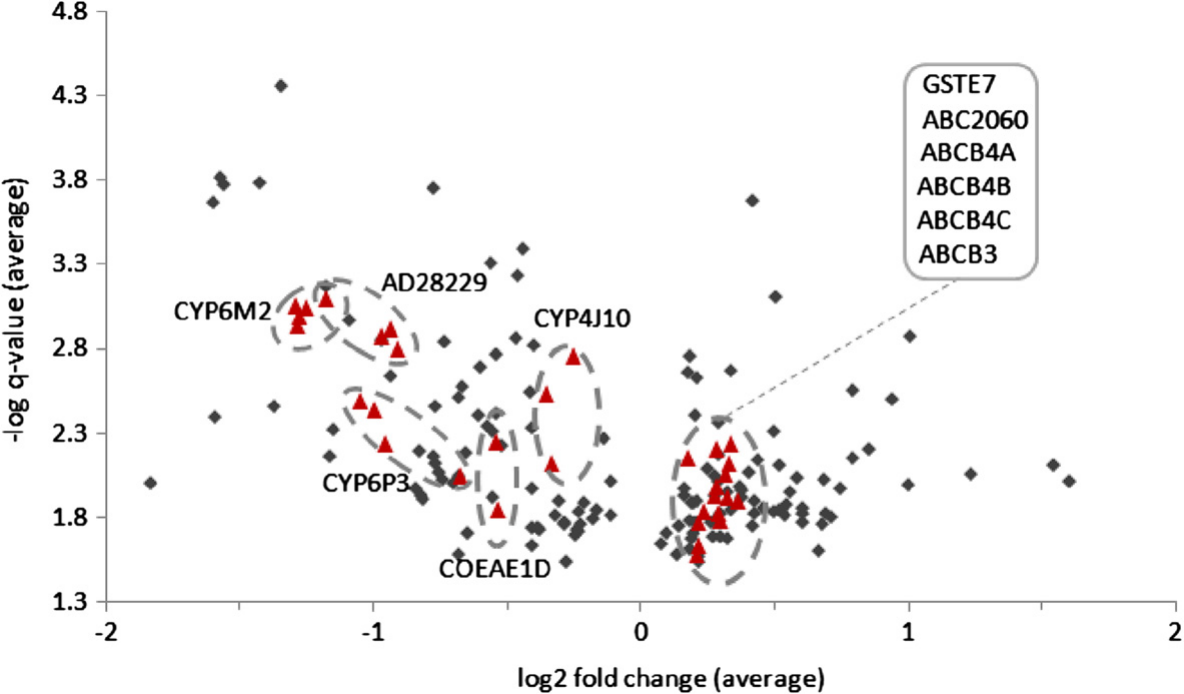
**Volcano plot of all probes significant in all of three experimental comparisons of *An. arabiensis *permethrin-exposed survivors *vs. *unexposed samples (average of the three experiments).** Red triangles show probes of candidate genes chosen for subsequent qRT-PCR, with probes for the same gene within dashed ovals; the tight cluster of overexpressed gene probes are grouped together.

**Figure 3:**
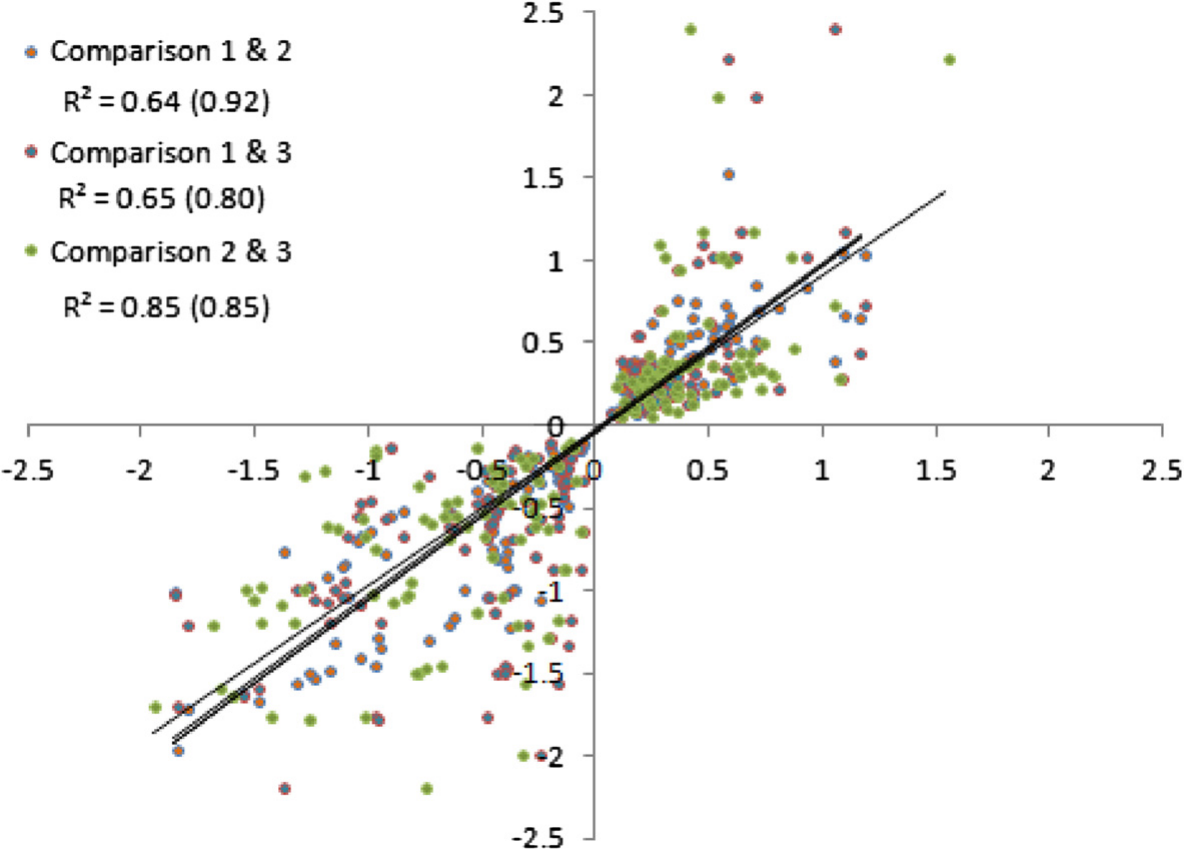
**Scatterplot illustrating the consistency of expression (measured as log**_**2 **_**fold change) for significant probes among the three experiments.** R^2^ value indicates all data; value in parenthesis the R^2^ value for genes selected for subsequent qRT-PCR.

**Figure 4:**
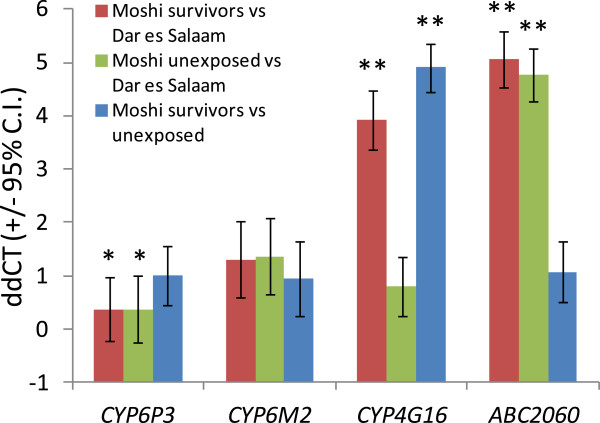
**Relative expression levels of four candidate genes between sample groups of *An. arabiensis *differing in predicted susceptibility.** Data are delta-delta CT values calculated from 5–9 biological repeats. **P < 0.01; *P < 0.05 (t-tests).

Microarray experiments involving Moshi samples indicated that 87 probes were significantly up regulated in all three comparisons and 85 down-regulated coming from a total of 133 genes (Figure 2). There was a high correlation among fold changes between the different experimental comparisons, suggesting consistency of results (Figure 3). Surprisingly, the genes that were down regulated include multiple detoxification genes, including several that have come up as top upregulated hits in previous *An. gambiae s.s.* arrays, including P450s repeatedly resistance-associated in previous work on *An. gambiae s.s.* (*CYP6M2* and *CYP6P3*). Among the up-regulated genes were 3 independent ABC transporters, one of which *ABCB4* (represented by 3 alternate transcripts) exhibited almost all significant probes. Also 3 probes for GSTe7 were up-regulated. Full microarray results from both experiments have been submitted as Additional file 1. However, few genes were selected for qPCR. Of about thirty primers that were designed, only six passed the criteria for qPCR. These primers are shown in Table 1 below including the primers for CYP4G16, the gene that was documented in other studies reporting on insecticide resistance in *An. arabiensis* and two normalising ‘control’ genes, ribosomal S7 and elongation factor EF1 (Table 1). Only two genes, ABC 2060 and CYP4G16 were the most significantly over-transcribed genes in qPCR (Figure 4).

**Table 1:** Primers used in quantitative real-time PCR (qPCR) (F = forward, R = reverse)

**Primer**	**Primer sequence**	**Fragment length (bp)**	**Reference**
ABC 2060	F: 5’-AATGCACTGCTTTGCGAACT- 3’	90	This paper
	R: 5’-GACCATCCCACTGTTTCGGA- 3’		
CYP6P3	F: 5’-GTGATTGACGAAACCCTTCGGAAGT-3’	N/A	Witzig *et al.,* 2013 [52]
	R: 5’-GCACCAGTGTTCGCTTCGGGA-3’		
CYP6M2	F: 5’-TACGATGACAACAAGGGCAAG- 3’	N/A	Witzig *et al.,* 2013 [52]
	R: 5’- GCGATCGTGGAAGTACTGG-3’		
CYP4G16	F: 5’- AGCTGAACGGATACCTGGACCGA-3’	81	Jones et *al.,* 2013 [40]
	R: 5’- AACACGGAGTGCGAACTGCCAAC-3’		
S7	F: 5’- AGAACCAGCAGACCACCATC-3’	149	Jones et *al.,* 2013 [40]
	R: 5’- GCTGCAAACTTCGGCTATTC-3’		
EF	F: 5’- GGCAAGAGGCATAACGATCAATGCG-3’	N/A	Jones *et al.,* 2013 [40]
	R: 5’- GTCCATCTGCGACGCTCCGG-3’		

## Discussion

Transcriptional analysis of pyrethroid resistance in the four day old Lower Moshi *An. arabiensis* females was investigated using the *An. gambiae* whole genome microarray [25] and quantitative real-time qPCR. Since a fully sequenced genome for *An. arabiensis* was not available; we used heterologous hybridization of the *An. arabiensis* samples to an *An. gambiae* microarray. The microarray analysis of this study clearly indicates that the differences among arrays could not be appropriately determined. In the previous studies, the use of *An. gambiae* ‘detox chip’ could successfully determine the gene expression of pyrethroid resistant populations of other species including *An. funestus *[53], *An. stephensi *[54] and *An. arabiensis *[28]. Gene expression of pyrethroid resistant *An. arabiensis* populations from Pemba Island was also successfully determined by using *An. gambiae* whole genome microarray [40]. The microarray analysis of this study clearly indicates that there were some very interesting hits among the up regulated genes most notably three independent ABC transporters and GSTe7.

Transcription of four genes was evaluated in this study using quantitative real-time (qPCR). It included one ABC transporter gene ABC 2060 that was up regulated, two P450s (CYP6M2, CYP6P3) that were down regulated in microarray experiments but previously associated with pyrethroid resistance and one P450 gene CYP4G16 that was identified through published literature and implicated in permethrin resistance in *An. arabiensis *[40]. Only two genes, CYP4G16 and ABC 2060, showed high expression levels (p < 0.05) in the pyrethroid resistant Lower Moshi *An. arabiensis* and it is possible that the two genes may be playing a role in the observed resistance to pyrethroids in the Lower Moshi *An. arabiensis* population. The low levels of expression of the two P450s, CYP6M2 and CYP6P3 in the pyrethroid resistant Lower Moshi *An. arabiensis* implies that the observed pyrethroid resistance results from other mechanisms rather than insecticide metabolism.

CYP4G16 has previously been recorded as an over-transcribed gene in *An. arabiensis* strain from Cameroon [28], Pemba Island Zanzibar [40] and in a laboratory strain of *An. arabiensis* from Sudan that was resistant to DDT, permethrin and deltamethrin [41]. In Pemba Island up-regulation of *CYP4G16* in resistant *An. arabiensis* ranged from 2.0 to 4.5 fold compared to susceptible strains of *An. arabiensis* from Dar es Salaam and Unguja and it has been suggested that it plays a role in cuticular-based resistance. Cytochrome P450s CYP4G17 and CYP4G16 of the *Anopheles gambiae* are closely related to the *Drosophila melanogaster* CYP4G1, the specific insect P450 with decarbonylase function that is involved in hydrocarbon biosynthesis. CYP4G16 and CYP4G17 proteins are found in the mosquito head and the carcass of the abdomen, MozAtlas [55] but not the midgut or malpighiam tubules, where P450 detoxification genes have been localized previously.

In this study, metabolic resistance to pyrethroids in Lower Moshi *An. arabiensis* has been shown to be associated with increased transcription of P450 gene, CYP4G16 and ABC transporter. Following qPCR analysis, CYP4G16 was over-expressed in Lower Moshi pyrethroid resistant *An. arabiensis* compared to susceptible strains of *An. arabiensis* from Dar es Salaam. (FC = 3.92, p = 0.007). The gene ABC 2060 was over-expressed with the FC of 5.07, (p = 0.009). ABC transporters have been linked to insecticide resistance in several species such as *Helicoverpa armigera *[56], *Aedes spp*. [57,58], *Culex pipiens* complex [59] and *Anopheles gambiae *[60]. ABC transporters have also been correlated with insect resistance to *Bacillus thuringiensis *[61]. Members of the ABC-transporter family (ABC-transporters) pump foreign molecules out of insect cells using an ATP-dependent mechanism [62-65]. They are also involved in lipid transport to the cuticle [62]. Interestingly, AGAP002060 is a member of the ABCH family, which have been implicated in transportation of lipids to the cuticle [62]. The previous study on *An.arabiensis* populations a few kilometers from Mabogini village [45] (the current study site) revealed over-transcription of esterase AGAP006227, the UDPGT AGAP006775 and the cytochrome P450s CYP9J4 and CYP6P1. However, its involvement in insecticide resistance is not clear. In addition, the gene AGAP000987 encoding the cuticle protein CPAP3-A1b was also strongly over-transcribed in *An. arabiensis* in the area. Cuticle proteins play an important role in pyrethroid resistance through altered insecticide penetration and over-transcription of genes encoding them have been reported in pyrethroid resistant species including *Anopheles species *[45,66].

## Conclusions

The study compared the gene expression profiles of permethrin-resistant and susceptible populations of *An. arabiensis* using quantitative real-time PCR (qPCR). *CYP6P3* is one of the leading candidate genes for metabolic resistance in West African *An. gambiae* and its down regulation suggests that quite different mechanisms may operate in the different species.

Based on the quantitative real-time PCR q(PCR), one P450 monooxygenase gene CYP4G16 and ABC transporter ABC 2060 were over-transcribed in permethrin resistance in Lower Moshi *An. arabiensis*. However, further investigation is needed to clarify its role in observed resistance. There is no real evidence that ABC 2060 transporter gene is linked to observed resistance, although significant lower expression of this gene was observed in a susceptible strain from Dar es Salaam than the Moshi population (i.e. either survivors or Moshi control). However, the two genes identified are most likely not the only genes involved in pyrethroid resistant *An. arabiensis* from Lower Moshi. Further investigation is needed especially in microarray analysis to identify more genes that may be involved in insecticide resistance in the country and its effects on the current malaria control interventions.

## Abbreviations

ITNs: Insecticide-treated nets; LLINs: Long-lasting insecticide treated nets; IRS: Indoor residual spraying; DDT: Dichlorodiphenyltrichloroethane; OPs: Organophosphates; GSTs: Glutathione S-transferases; P450: Cytochrome P450 monooxygenase enzymes; RNA: Ribonucleic acid; qPCR: Quantitative Polymerase Chain Reaction; RT-qPCR: Reverse transcription and quantitative polymerase chain reaction; cDNA: Complementary Deoxyribonucleic acid; CYP: Genes encoding cytochrome; NCBI: National Center for Biotechnology Information; ANOVA: Analysis of variance; MAANOVA: Multivariate Analysis of Variance; MCDC: Malaria Capacity Development Consortium; KCMC: Kilimanjaro Christian Medical Centre; WHO: World Health Organization.

## Competing interests

The authors declare that they have no competing interests.

## Authors’ contributions

JM conceived, designed, and performed the experiments; participated in data analysis and interpretation; wrote the first draft of the manuscript. CMJ participated in RNA extractions, labelling, hybridizations and scanning of the microarrays; contributed to data analysis and interpretation and reviewed the manuscript. BK contributed to sampling, processing and preservation of samples and review of the manuscript. KS processed mosquitoes and performed reverse transcription and qPCR. FM and MR critically revised the manuscript. HR provided technical support and facilitated the implementation of the microarrays work. DW co-designed the study, provided technical support, contributed to data analysis and interpretation and critically reviewed the manuscript. All authors have read and approved the final manuscript.

## Supplementary Material

Additional file 1 Full microarray results from both experiments. Click here for file
